# Supplementary material for: Comparison of analytical sensitivity of DNA-based and RNA-based nucleic acid amplification tests for reproductive tract infection pathogens: implications for clinical applications
Source: Microbiol Spectr. 2023 Aug 22;11(5):e01497-23. doi: 10.1128/spectrum.01497-23 (PMC10581061; doi:10.1128/spectrum.01497-23)
Supplement: Supplemental information — Tables S1 to S3. [file spectrum.01497-23-s0001.pdf]

Supplementary material

Table S1 Sequences of synthetic plasmids

Plasmid	Synthetic sequence	Length
Barcode plasmid	barcode sequence	259 bp
CT plasmid	barcode sequence and 23S rRNA of *CT* (NC_000117.1)	259 bp + 2870 bp
NG plasmid	barcode sequence and 16S rRNA of *NG* (NR_026079.2)	259 bp + 1541 bp
UU plasmid	barcode sequence and 16S rRNA of *UU* (NC_011374.1)	259 bp + 1513 bp

Abbreviations: *CT*, *Chlamydia trachomatis*; *NG*, *Neisseria gonorrhoeae*; *UU*, *Ureaplasma urealyticum*. The barcode sequence here was a 259 bp long non-coding RNA of *Arabidopsis thaliana*:

AGGGGTGTGGGAACCTAGGAGATGAGTCTGCTTATTGATTGCTAAACCCTG
AACCCTCTCATGTTAACTATGGGAATTAATTACTGGGGGTCTTAGGCCGGCG
TTTCCCCCGAAAAATAATGAAAAAACGTCGATGGCTACATAAGAGGCTCGT
CTCCAAAAAGTAGACCAGGAGGTTGGGTTTGGTTCGTAGGTGGTTCTGTTG
AAACTAGATTAGTGTCTGCGGTTAACCGTTTCCTCGCCTTACCCTCCCACCC
CC

Table S2 The primers and probes for DNA and RNA quantification

Plasmid/Pathogen	Target gene	Primer/Probe sequence (5' to 3')
Barcode plasmid	barcode sequence	QF：TTAATTACTGGGGGTCTTAGGC
		QR：CACCTACGAACCAAACCCAA
		QP：6-FAM-ACGTCGATGGCTACATAAGAGG-BHQ1
CT plasmid and *CT*	23S rDNA	QF：TCCCTCGCCGTAAGCCCAAG
		QR：TTTAACCTGCTGCTCCATCGT
		QP：6-FAM-CCAGGGTCAAGCTCGTCTTCC-BHQ1
NG plasmid and *NG*	16S rDNA	QF：CAGCTAATACCGCATACGTCT
		QR：GCTACTGATCGTCGCCTTGGTG
		QP：6-FAM-CCTTTACCCCGCCAACCAGCTA-BHQ1

UU plasmid and *UU*	16S rDNA	QF：CCCTAGTAGTCCACACCGTA<br>QR：CCGTCAATTCCGTTTGAGTT<br>QP：6-FAM-ATGTGCCTGGGTAGTACATTCG-BHQ1

Abbreviations: *CT*, *Chlamydia trachomatis*; *NG*, *Neisseria gonorrhoeae*; *UU*, *Ureaplasma urealyticum*.

Table S3 Amplification efficiency of qPCR

Plasmid	Quantification curve	R^2	Amplification efficiency	Copy number ratio of target/barcode±standard deviation
Barcode plasmid	y = -3.2451x + 44.878	0.9989	103%	/
CT plasmid	y = -3.1831x + 45.298	0.9994	106%	1.067±0.11
NG plasmid	y = -3.2231x + 42.924	0.9983	103%	1.174±0.163
UU plasmid	y = -3.3236x + 42.477	0.9952	99.9%	1.076±0.085

Abbreviations: *CT*, *Chlamydia trachomatis*; *NG*, *Neisseria gonorrhoeae*; *UU*, *Ureaplasma urealyticum*; R^2, coefficient of determination. These ratios were obtained from six replicates of ddPCR arrays.
